# Editorial expression of concern: Cdc6 expression is induced by HPV16 E6 and E7 oncogenes and represses E-cadherin expression

**DOI:** 10.1038/s41417-020-0165-z

**Published:** 2020-02-06

**Authors:** E. Faghihloo, M. Sadeghizadeh, S. Shahmahmoodi, T. Mokhtari-Azad

**Affiliations:** 1 Department of Microbiology, School of Medicine, Shahid Beheshti University of Medical Sciences, Tehran, Iran; 2 Department of Genetics, Faculty of Biological Sciences, Tarbiat Modares University, Tehran, Iran; 3 Department of Virology, School of Public Health, Tehran University of Medical Sciences, Tehran, Iran

**Addendum to:Cancer Gene Therapy**

10.1038/cgt.2016.51

After careful review and discussions with the authors, the Editor-in-Chief is issuing an editorial expression of concern to alert readers that the cell lines used in this study [1] may have been contaminated. The data reported in this article should therefore be interpreted with caution. All authors agree with this notice.
